# Dr. Thackeray, on Cold Water in Convulsions

**Published:** 1803-11-01

**Authors:** James Currie

**Affiliations:** Liverpool


### To Dr. BATTY.

DEAR SIR,

THE enclosed Case was communicated to me by Dr. Thackeray. I think it well deserves to be laid before the public; and if you agree with me, I beg you will insert it in the Medical and Physical Journal.

*Liverpool,* I am, &c.

*August* 10, 1803. JAMES CURRIE.

---

On the 10th of November last I was called to see a young lady in Shropshire, who had been attended by Mr. Cartwright, a respectable surgeon and apothecary at Oswestry, whose case was as follows. M. V. æt. 18, with light hair and a pale complexion, had been some time subject to affections in the stomach and bowels, indigestion, flatulence, costiveness, great oppression at the præcordia, nausea, with occasional vomiting and singultus. Her catamenia had been profuse, and followed by fluor albus. All these symptom had increased and been accompanied by spasmodic affections, at length arising to general convulsions. During these convulsions the belly was for a moment soft and again hard and contracted, and the spasms extended to the thighs and legs, arms, back, and neck. These general convulsions occurred four or five times every day. She had besides an irritating cough, dyspnœa, flushings, and night sweats. She was become very feeble, and seemed declining rapidly. I first ordered an emetic, and afterwards various antispasmodics, as bark, camphire, asafœtida, opium, and the warm bath. She had besides

2 fomen fomentations and clysters. These procured some temporary relief.

I was sent for again on the 18th, and all the symptoms were increased. The cough was more troublesome with considerable expectoration, and the chills and flushings more frequent. In addition to the antispasmodic medicines, the digitalis was prescribed with castor; one grain of the powder was ordered every four hours, and the dose was at length augmented to two grains and a quarter. The pectoral and spasmodic affections were both strikingly relieved, but sickness, vertigo, and the other peculiar effects of digitalis coming on, it could not be continued.

I was sent for again on the 30th, and found all the complaints aggravated. The spasmodic affections were now constant and exhausting, and the general convulsions occurred more frequently than ever, were accompanied by the abolition of consciousness, and followed by delirium. The spasm extended to the bladder, and occasioned great distress. She complained particularly of burning heat in the sockets of the eyes, and acute pain of the whole tongue, both of which were somewhat relieved by pressure. Her stomach rejected every thing, cordials as well as the simplest food. She was extremely emaciated and unable to stand upright. All the usual remedies being exhausted, and the case appearing dangerous in the extreme, I determined to try the affusion of cold water, as recommended by Dr. Currie, of Liverpool. She had been affected by vomiting for upwards of half an hour, with little or no discharge; the abdomen was hard and contracted; there was considerable dyspnœa; the pulse was rapid and irregular, the skin cool and rather clammy; shiverings had come on, terminating in general convulsions and insensibility, under which she was lifted out of bed and placed under the cold-shower bath, containing nine gallons of water, her head having been previously shaved to increase the impression. The water descended upon her and in a moment her consciousness returned and the convulsions disappeared. Her pulse sunk to 80, her breathing became regular, and her stomach was found to bear nourishment. She became quiet and composed. In the course of the day the convulsions returned, and twice they were relieved in the same way by the cold affusion. They returned the third time at eleven at night, and the remedy now seemed to have lost its effect. She passed a restless night with repeated convulsions and vomiting. About eight in the morning she was however sensible, and she said she thought the failure of the bath bath the last time of trial had been owing to its not being used properly, the water having fallen upon her shoulders, and not on her head. She desired to have it tried again, and in a full stream. Her directions were followed; the water descended suddenly and profusely on her head during the next general convulsion, and again the symptoms vanished and consciousness was restored. She now fell into a quiet sleep, which lasted several hours, and she awoke from it wonderfully refreshed and composed. Some slight partial spasmodic affections re-appeared, but the general convulsions have never returned, and she has increased in strength every day. The cold affusion was directed to be repeated morning and evening, which is still continued, and two pills were directed to be given thrice a day, containing at each dose half a grain of cuprum ammoniacale, the same quantity of opium, three grains of calcined zinc, and four grains of the extract of cicuta. I ascribe the recovery however entirely to the cold affusion used during the existence of general convulsion. Written January 24.

February 12, 1803. For a month from the 30th of December, she continued well and had recovered her flesh. She persevered in the bathing night and morning, and drank a pint of wine daily.

On the 27th of January the catamenia appearing, they discontinued the bath. Sitting in the evening with her family she was suddenly taken as before, with general spasms, dyspnœa, vomiting, and delirium. In this state they put her under the shower-bath thrice, which instantly removed her complaints, but the effect was transitory.

On the 28th at twelve at night, I saw her, and found her in the same state as formerly, but with delirium. Her situation was in some respects very singular. She would sit up in bed with twitchings all over her, particularly affecting the head, &c. and repeat passages of poetry, sometimes losing a word, but not recovering it when suggested to her, or appearing sensible that the suggestion was made. This continued several times. She also wrote a letter to a friend, directed it, and enclosed it to a peer. Some hours after, remaining in the same state, she read a favourite paper in the Rambler, and got part of it by heart. When she found herself perfect, she desired me to hear her repeat it. She gave me the entire sense, but altered many of the words. I then ordered thirteen gallons of iced water to be dashed over her at once; but though it brought her to her senses, she said it did not produce the same relief as the shower-bath, nor was it followed by the same comfortable glow.

glow. We therefore employed the shower-bath that night four times in all, and every time with sudden and striking advantage, the patient recovering her senses in a moment. The last time was at three o'clock on Sunday morning; at ten, eleven, and one, she took three draughts with 70 drops of tinct. opii, and 25 of tinct. digitalis in each, without the least apparent effect. No sleep. Sickness, vomiting, and spasms continued on Sunday morning.

The shower bath was repeated twice or thrice a day, and after the fourth day at noon no spasms or convulsions returned.

*The Case continued by Miss V.*

" From the time you quitted us, she had no spasmodic return till the 14th of March, when her head was dreadfully afflicted.

" In the night of the 16th she felt an uncontrolably ravenous appetite, though no aliment taken at the time would remain five minutes on her stomach; this craving returned at the same hour each succeeding night until the 21st, after which it never affected her in any degree, (that night she took the first of the three doses of calomel you ordered).

" On the 26th, 28th, and 29th, she had repeated and severe spasms in her head; in every instance the shower-bath, (and she used it fourteen times in twenty hours) has infallibly afforded instantaneous though temporary relief.

" On the 31st, symptoms of the influenza, confirmed on the 2d of April, and attended by a spasm in the head.

" April 9, all effects of the influenza had nearly disappeared, her spirits, strength, appetite, sleep, all seemed restored to their wonted state.

" Immediately on awaking in the morning of the 26th, a dreadful attack in her head, which recurred daily till the 29th, with the most aggravated and distressing symptoms; sudden stupors, from which she was roused with the greatest difficulty, and during which her countenance was drawn and distorted with involuntary motion; great alienation of mind without any previous complaint of pain; these alarming appearances subsided gradually by the repetition of the bath, and she had no recurrence of spasm till the night of the 9th of May, which attack may be imputed to the irritation occasioned by a journey of nearly forty miles, and a previous sleepless night. (She was on her road to Abergally.) She had a slight return on the morning of the 10th, another on the 15th, a much severer one on the

24th

24th, two on the 25th, but unattended with stupors or alienation of mind, being able each time to walk down one flight of stairs to the shower-bath, which always had the usual good effect.

"In the night of the 29th, a very violent spasm.

"On the 30th, two fainting fits, great oppression at her chest.

"On the 1st of June, a dreadful attack in her head and stomach; delirium and convulsions, without sleep, till the 6th."

I was with her on the 4th, and did not leave her room till the morning of the 6th. From Sunday at eight in the evening till four the next morning she took 350 drops of laudanum, 9 grains of opium, and 85 drops of tincture of digitalis, *without the least effect.* The shower-bath alone checked the convulsions and brought her to her senses, and we were compelled to have recourse to it once, twice, or oftener in an hour. It required three people to take her down stairs; but the moment she was placed in the tub, she became quiet and clasped her knees with her hands. As soon as the water touched her head, she exclaimed, "I am well." Clothes dipt in a strong solution of ammonia muriatis, and applied to the head, were of great use.

On the 10th she had a most violent diarrhœa, which continued till the following evening. This was easily removed, and she is now in good health.

